# Motor learning strategies in basketball players and its implications for ACL injury prevention: a randomized controlled trial

**DOI:** 10.1007/s00167-015-3727-0

**Published:** 2015-08-11

**Authors:** Anne Benjaminse, Bert Otten, Alli Gokeler, Ron L. Diercks, Koen A. P. M. Lemmink

**Affiliations:** 1 Center for Human Movement Science, University Medical Center Groningen, University of Groningen, Antonius Deusinglaan 1, 9713 AV Groningen, The Netherlands; 2 School of Sport Studies, Hanze University Groningen, Zernikeplein 17, 9747 AS Groningen, The Netherlands; 3 Center for Sports Medicine, University Medical Center Groningen, University of Groningen, Postbus 30.001, 9700 RB Groningen, The Netherlands

**Keywords:** ACL, Injury prevention, Motor learning, Attentional focus

## Abstract

**Purpose:**

Adding external focus of attention (EF, focus on the movement effect) may optimize current anterior cruciate ligament (ACL) injury prevention programmes. The purpose of the current study was to investigate the effects of an EF, by a visual stimulus and an internal focus, by a verbal stimulus during unexpected sidestep cutting in female and male athletes and how these effects remained over time.

**Methods:**

Ninety experienced basketball athletes performed sidestep cutting manoeuvres in three sessions (S1, S2 and S3). In this randomized controlled trial, athletes were allocated to three groups: visual (VIS), verbal (VER) and control (CTRL). Kinematics and kinetics were collected at the time of peak knee frontal plane moment.

**Results:**

Males in the VIS group showed a larger vertical ground reaction force (S1: 25.4 ± 3.1 N/kg, S2: 25.8 ± 2.9 N/kg, S3: 25.2 ± 3.2 N/kg) and knee flexion moments (S1: −3.8 ± 0.9 Nm/kg, S2: −4.0 ± 1.2 Nm/kg, S3: −3.9 ± 1.3 Nm/kg) compared to the males in the VER and CTRL groups and to the females in the VIS group (*p* < 0.05). Additionally, the males in the VIS group reduced knee valgus moment and the females in the VER group reduced knee varus moment over time (n.s.).

**Conclusion:**

Male subjects clearly benefit from visual feedback. Females may need different feedback modes to learn a correct movement pattern. Sex-specific learning preferences may have to be acknowledged in day by day practice. Adding video instruction or feedback to regular training regimens when teaching athletes safe movement patterns and providing individual feedback might target suboptimal long-term results and optimize ACL injury prevention programmes.

**Level of evidence:**

I.

## Introduction

Although current ACL injury prevention programmes are effective [50], success is highly dependent on compliance. Athletes with high compliance have significantly reduced ACL injury rates compared to athletes with low compliance [50]. Coaches are hesitant to implement as they may feel it is ‘too much’ and not of their primary interest [1, 16, 21, 27, 32, 38]. Hence, there is a clear need to improve the efficiency of ACL injury prevention strategies [50]. A novel approach in ACL injury prevention would be to adopt knowledge of motor learning [4, 6]. A common denominator of current programmes is the use of explicit instructions and feedback regarding desired landing positions [39]. Motor skills can be learned with an internal focus of attention (IF, focus on the movement themselves, e.g. ‘flex your knees when landing’) or with an external focus of attention (EF, focus on the movement effect, e.g. ‘touch target as you land’) [55]. Learning strategies with an IF have been shown to be less suitable for the acquisition of complex motor skills required for sports [55], while EF enhances automatic motor control [55]. Compared to IF or no instruction, an EF attention has superior results on jump landing performance [7], with an improved transfer to sport [55].

For prevention, it is imperative to reduce knee joint loading during sport-specific tasks like sidestep cutting [25]. Kinematics and kinetics during sidestep cutting manoeuvres have been thoroughly examined [5]; however, no clear explanation for observed sex differences could be found. Dempsey et al. [14] examined the effect of feedback and used both visual (self and expert) and verbal (IF and EF) feedback, resulting in a reduced peak knee valgus moment in male athletes. Celebrini et al. examined female athletes and provided EF instructions directed to a styrofoam ball around the waist (focus on a point outside the body), resulting in increased peak knee flexion angles [8, 9]. In addition, retention (i.e. movement strategy used after a certain time interval when no feedback, guidance or instructions are given and all groups perform under the same conditions) is important as ideally, properly learned motor skills become relatively permanent [47] and therefore the newly learned skills need only periodic maintenance. However, the effect of IF and EF instructions on sidestep cutting kinetics and kinematics and retention of learned skills is unknown. Considering the sex differences often referred to in the literature, it is also imperative to know how females and males respond to stimuli given in terms of movement strategies. This information is clinically relevant as it can then be used to potentially incorporate sex-specific instruction and feedback strategies and optimize ACL injury prevention programmes with long-term effects.

The purpose of this study was therefore to investigate the effects of a visual EF and a verbal IF feedback on peak knee joint loading [15] during unexpected sidestep cutting over time in female and male athletes. We hypothesize that the athletes, regardless of sex, receiving visual feedback (VIS) reduce knee joint loading with better retention compared to the athletes in the verbal feedback (VER) and control (CTRL) groups.

## Materials and methods

A randomized controlled trial was conducted in a controlled laboratory setting. Ninety healthy recreational basketball players recruited from local clubs were included (45 females, 22.3 ± 3.7 years, 175.9 ± 6.7 cm, 67.8 ± 8.4 kg; 45 males, 24.9 ± 4.6 years, 190.9 ± 6.7 cm, 82.3 ± 8.5 kg). Enrolment, allocation and testing were conducted by the first author. Inclusion criteria were: ≥18 years and playing at highest recreational level (practice 3 times a week for ≥2 h). Subjects were excluded if they had a history of lower extremity injury or surgery in the 6 months prior to testing. Based on the order of inclusion, subjects were randomly assigned (stratified for sex) to one of the three groups: VIS, VER or CTRL (no feedback).

### Procedures

Informed written consent was obtained prior to inclusion. First, anthropometric measures were taken prior to placement of 21 reflective markers of 14 mm in diameter placed according to the Vicon Plug-in-Gait marker set, with additional trunk markers on the sternum, clavicle, C7, T10 and right scapula. This was followed by a static calibration. All subjects wore spandex shorts and shirts (for females) and their own athletic shoes. The tasks chosen for this experiment were a 45° sidestep cut, straight run or 45° crosscut, which were randomly indicated with a green light. The straight run and 45° crosscut were used as additional tasks in order to present the subject with three options (sidestep cut, run and crosscut) [3, 41, 42]. Subjects used a 5-m approach run followed by a 1-foot landing on the force plate and a 45° change in direction through a second set of timing gates 5 m away from the force plates. To reassure standardization while challenging athletes, both approach and exit speed had to be within 4.5–5.5 m/s. Subjects were instructed to land on the force plate with their dominant leg, which was defined as the leg they prefer pushing off with while jumping. The general instruction for all groups was: ‘run towards the force plate and just before the force plate you will see a bright green light showing up, indicating the direction you have to run after placing your foot on the force plate. After passing the forces plate continue running until you have passed the timing gates’. Each subject was given ample time to warm up and familiarize themselves with the set-up.

After familiarization, the first three trials served as a baseline. After baseline collection, the first session (S1) was started, in which feedback was provided to the VIS and VER groups after every correct trial. In S1, the VIS group received video feedback on a TV screen (LG, Flatron 65VS10-BAA) showing the subject from behind. A new video was only shown if the subject performed better than the previous best trial; this became the new best trial (i.e. smaller external peak knee frontal plane moment). No explicit feedback or instructions were given; however, subjects knew that they were looking at their best trial so far. They were instructed to replicate that trial to the best of their ability. In SI, the VER group received the following instructions: (1) ‘bend your trunk forward’, (2) ‘bend your knee’ and (3) ‘keep your knee straight above your foot’. The CTRL group was only provided with the general instructions. Two retention sessions were conducted, after 1 week (S2) and 4 weeks (S3). No feedback at all was given during the retention tests.

Thirty-five successful sidestep cutting trials, defined as correct speed and cut angle of 45° (marked with tape) with the correct foot on force plate, were collected per session. Subjects were aware of the location of the force plate, but as they were focusing on the light stimuli, targeting of the plate was avoided. Also, tape was placed at the start of the approach distance to facilitate the desired foot contacting the force plate. Trials were rejected if the subject clearly targeted the plate (i.e. a ‘stutter step’ or ‘reaching’). Each subject was given enough rest between trials to reduce the potential effects of fatigue.

### Apparatus

Kinematic data were collected using an 8 camera motion analysis system at 200 Hz [Vicon Motion Analysis Systems Inc., Oxford, UK and Vicon Nexus software (version 1.8.3, Oxford, UK)]. Good measurement accuracy and high test and retest repeatability have been previously reported [22, 31]. Ground reaction force data were collected at 1000 Hz with two Bertec force plates (Bertec Corporation, Columbus, OH). To provide feedback to the VIS group, a Basler camera (640 × 480, 210 fps, Vicon Motion Systems, Inc., Centennial, CO) with a 25-mm C-mount lens was used to collect analogue high-speed data. Two infrared timing gates (HL 2-31 Photocell, TAG Heuer professional timing, Switzerland) were used to ensure that running speed was 4.5–5.5 m/s. A 3-light guiding system was used to randomly cue the subject 0.5 s before stepping on the force plate [34].

Procedures were approved by the University of Groningen Medical Ethics Committee (ID numbers: CCMO protocol number: NL24814.042.09, METc: 2009.142).

### Data acquisition and statistical analysis

Based on previous research [34, 41, 48], sample size was estimated for a minimal statistical power of 80 % (*α* = 0.05). All sample size and power calculations were completed using (G*Power for Mac, version 3.1.2). Given the variation of the dependent measures that were included in this study, 15 subjects per group (male and female) were deemed adequate. Hence, 15 females and 15 males were allocated to the VIS, VER and CTRL groups, respectively.

Primary outcome variables were vertical ground reaction force (vGRF), sagittal angles and moments of the trunk, hip, knee, ankle and range of motion (RoM). For the knee, frontal plane moments were also collected. All variables are expressed at peak external valgus/varus moment. RoM was calculated as the value at peak external valgus/varus moment minus the value at initial contact. Moments are expressed as external moments normalized to body weight. Results in degrees will be reported to one decimal case [53]. Based on number of subjects and standard deviation, effect sizes (ESs) were calculated for all comparisons. Cohen’s *d* values are reported as a measure of ES, where 0.2 ≤ *d* ≤ 0.5, 0.5 ≤ *d* ≤ 0.8 and *d* ≥ 0.8 represent a small, moderate and large effect, respectively [11].

Sidestep trials were analysed of all included subjects (*n* = 30 VIS, *n* = 30 VER, *n* = 30 CTRL). Customized software using MATLAB 6.1 (The MathWorks Inc., Natick, MA) was written and used to compute segmental kinematics and kinetics of the tested leg. Force plate and kinetic data were filtered using a fourth-order zero-lag Butterworth low-pass filter at 10 Hz. Assumptions for normality of distribution for all variables were checked. Assumptions of homogeneity of variance and sphericity were also validated for the use of analysis of variance (ANOVA). Differences between groups at baseline were determined using a multivariate ANOVA. To determine differences between groups (VIS, VER, CTRL), time (S1, S2, S3) and sex (female, male), a 3 × 3 × 2 repeated measures ANOVA was conducted followed by post hoc comparisons (Bonferroni) with alpha level set at *α* ≤ 0.05 a priori. Additionally, the start and end values of each session were calculated (linear fit of all data).

## Results

**Fig. 1:**
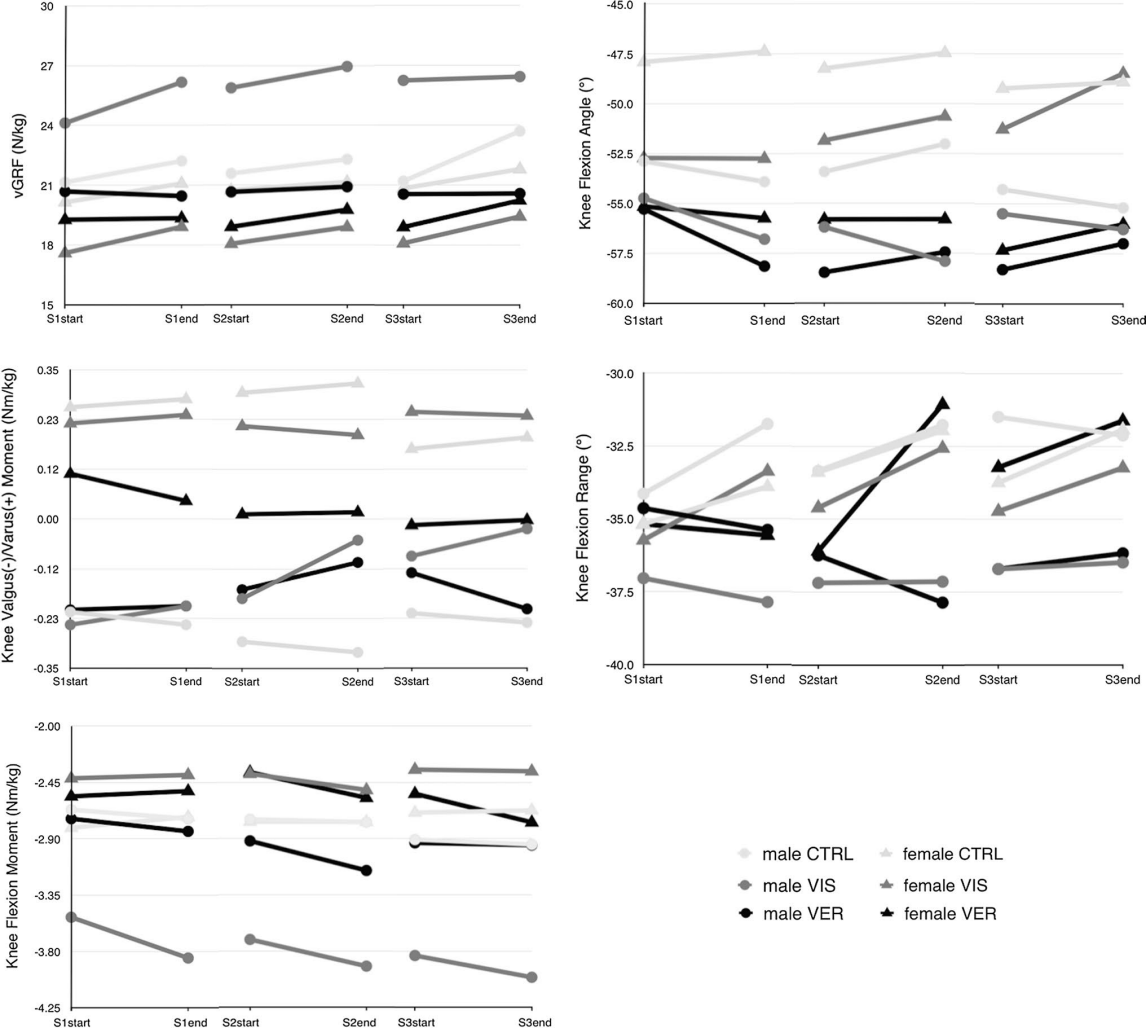
Group changes in kinetics (*left column*) and kinematics (*right column*) in S1 and retention sessions (S2 and S3)

At baseline, except for ankle dorsiflexion angle, no significant differences in kinematics and kinetics were found neither across the male groups (VIS, VER, CTRL) nor across the female groups (VIS, VER, CTRL) (n.s.). Results are presented in Figs. 1 (change over time per session) and 2. Detailed kinematic and kinetic results are presented in Tables 1 and 2, respectively (means of 35 trials per session, SD’s and 95 % CIs). Average approach speed was 5.0 ± 0.2 m/s, while average exit speed was 4.8 ± 0.2 m/s (no significant group, session or sex differences).

**Fig. 2:**
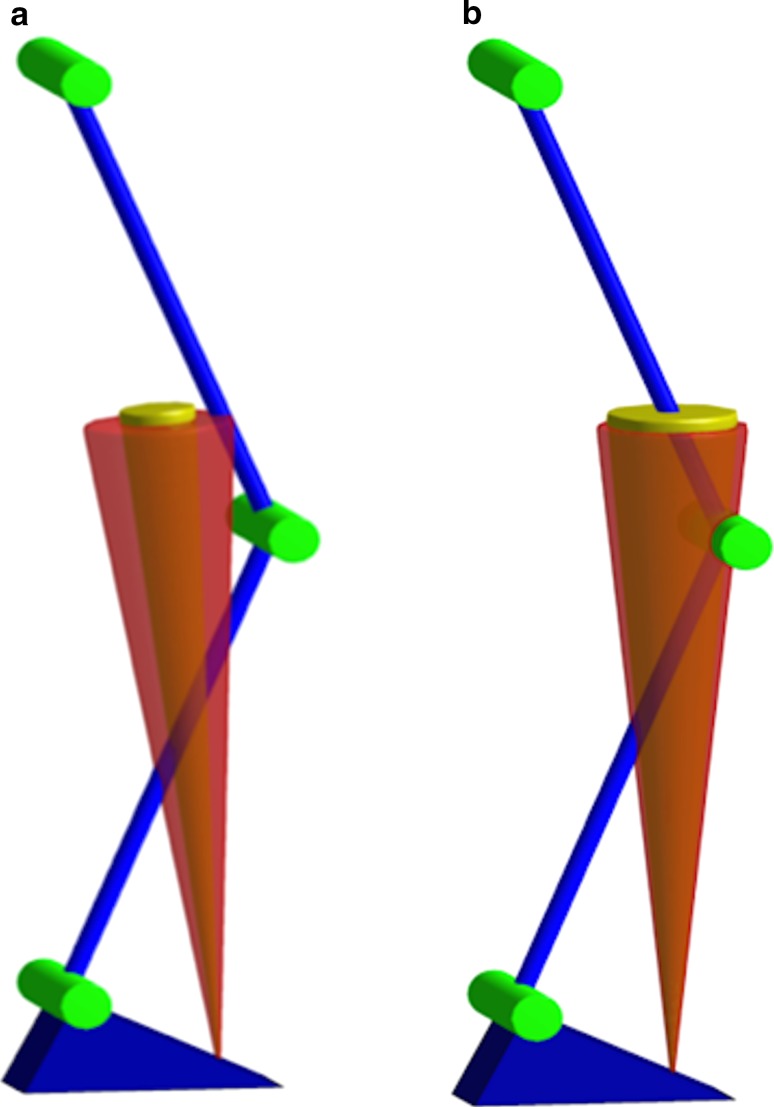
Schematic representation of collection of possible vGRF’s (*cone*) and knee flexion moment in **a** males and **b** females in the VIS group. *Red cone* represents baseline vGRF, and *yellow cone* represents vGRF after visual feedback. Note that males kept knee flexion moment high, while reducing knee valgus moment, whereas females did not

**Table 1:** Results of repeated measures ANOVA for the dependent kinematic variables

Kinematic variables	Sex	CTRL		VIS		VER		*p* value group	*p* value time	*p* value sex
		Mean ± SD	95 % CI	Mean ± SD	95 % CI	Mean ± SD	95 % CI			
Trunk flexion angle (°) S1	Male	11.0 ± 4.2	8.4–13.5	12.7 ± 7.4	8.6–16.8	12.3 ± 7.1	8.3–16.2	n.s.	n.s.	0.054 (CTRL) ES = 0.72
Trunk flexion angle (°) S2		10.9 ± 4.4	8.3–13.5	14.6 ± 8.9	9.7–19.6	13.4 ± 7.5	9.3–17.6	n.s.	n.s.	
Trunk flexion angle (°) S3		9.9 ± 4.8	7.0–12.8	13.3 ± 6.7	9.5–17.0	13.0 ± 6.3	9.6–16.5	n.s.	n.s.	
Trunk flexion angle (°) S1	Female	15.2 ± 5.5	11.8–18.5	11.8 ± 4.3	9.4–14.2	12.4 ± 4.3	10.0–14.8	n.s.	n.s.	
Trunk flexion angle (°) S2		14.7 ± 6.1	11.0–18.4	12.8 ± 4.6	10.3–15.3	12.8 ± 3.2	11.0–14.6	n.s.	n.s.	
Trunk flexion angle (°) S3		13.1 ± 6.0	9.5–16.8	14.2 ± 4.9	11.5–17.0	12.0 ± 3.5	10.0–13.9	n.s.	n.s.	
Hip flexion angle (°) S1	Male	24.7 ± 7.0	20.5–28.9	27.0 ± 13.5	19.2–34.8	30.3 ± 12.5	23.4–37.3	n.s.	n.s.	0.054 (CTRL) ES = −0.68
Hip flexion angle (°) S2		28.2 ± 11.2	21.4–35.0	25.8 ± 11.4	19.2–32.4	32.9 ± 12.4	26.1–39.8	n.s.	n.s.	
Hip flexion angle (°) S3		25.0 ± 8.1	20.1–29.9	25.7 ± 10.9	19.4–32.0	27.6 ± 13.5	20.2–35.1	n.s.	n.s.	
Hip flexion angle (°) S1	Female	19.9 ± 9.3	14.5–25.3	22.6 ± 10.0	17.0–28.2	24.0 ± 10.2	18.4–29.6	n.s.	n.s.	
Hip flexion angle (°) S2		20.1 ± 9.4	14.7–25.5	19.8 ± 11.5	13.5–26.2	23.2 ± 8.6	18.5–28.0	n.s.	n.s.	
Hip flexion angle (°) S3		20.2 ± 8.4	15.4–25.1	19.4 ± 12.6	12.4–26.3	23.3 ± 11.8	16.7–29.8	n.s.	n.s.	
Knee flexion angle (°) S1	Male	−53.4 ± 6.5	−57.1 to −49.7	−55.8 ± 5.3	−58.8 to −52.7	−56.7 ± 5.1	−59.7 to −53.8	n.s.	n.s.	0.047 (VIS) ES = −0.91
Knee flexion angle (°) S2		−52.7 ± 7.4	−57.1 to −48.6	−57.1 ± 4.8	−59.8 to −54.3	−58.0 ± 6.5	−61.7 to −54.2	n.s.	n.s.	
Knee flexion angle (°) S3		−54.7 ± 8.0	−58.8 to −49.6	−55.9 ± 6.1	−59.40 to −52.39	−57.7 ± 5.8	−61.4 to −54.7	n.s.	n.s.	
Knee flexion angle (°) S1	Female	−47.6 ± 6.0	−52.1 to 44.2	−52.7 ± 5.1	−55.53 to −49.94	−55.4 ± 5.6	−58.5 to −52.4	0.004 (VER vs. CTRL) ES = 1.35	n.s.	
Knee flexion angle (°) S2		−47.9 ± 5.3	−50.9 to −44.8	−51.2 ± 4.7	−53.85 to −48.64	−55.8 ± 5.0	−58.6 to −53.0	0.001 (VER vs. CTRL) ES = 1.53 0.012 (VER vs. VIS) ES = 0.94	n.s.	
Knee flexion angle (°) S3		−49.1 ± 6.5	−52.8 to −45.3	−49.9 ± 6.7	−53.57 to −46.18	−56.7 ± 4.6	−59.2 to −54.2	0.007 (VER vs. CTRL) ES = 1.35 0.003 (VER vs. VIS) ES = 1.19	n.s.	
Ankle dorsiflexion angle (°) S1	Male	4.8 ± 4.9	2.0–7.6	9.4 ± 3.4	7.4–11.5	7.4 ± 7.1	3.4–11.3	0.017 (VIS vs. CTRL) ES = −1.11	0.034 (CTRL S1 vs. S3) ES = −0.46 0.048 (VER S1 vs. S3) ES = −0.35 0.041 (VER S2 vs. S3) ES = −0.40	n.s.
Ankle dorsiflexion angle (°) S2		6.3 ± 4.2	3.9–8.7	11.0 ± 4.5	8.3–13.7	7.3 ± 5.7	4.1–10.4	0.018 (VIS vs. CTRL) ES = −1.08		
Ankle dorsiflexion angle (°) S3		7.1 ± 5.1	4.1–10.0	10.5 ± 5.7	7.1–14.0	9.6 ± 6.0	6.3–13.0	0.041 (VIS vs. CTRL) ES = −0.64		
Ankle dorsiflexion angle (°) S1	Female	5.8 ± 5.7	2.6–8.9	7.9 ± 5.5	4.4–11.4	8.3 ± 5.0	5.5–11.0	n.s.	0.052 (CTRL S1 vs. S2) ES = −0.34 0.016 (VER S1 vs. S3) ES = −0.61	
Ankle dorsiflexion angle (°) S2		7.8 ± 5.9	4.5–11.0	8.5 ± 8.1	3.4–13.7	9.8 ± 5.5	6.8–12.8	n.s.		
Ankle dorsiflexion angle (°) S3		7.6 ± 6.0	4.3–10.8	7.1 ± 5.3	3.8–10.5	11.3 ± 4.9	8.6–14.0	n.s.		
Hip RoM (°) S1	Male	7.3 ± 4.9	4.4–10.3	8.2 ± 5.2	5.2–11.2	7.0 ± 8.6	2.3–11.8	n.s.	n.s.	n.s.
Hip RoM (°) S2		6.3 ± 5.2	3.2–9.5	7.6 ± 6.7	3.8–11.5	7.3 ± 8.5	2.6–12.0	n.s.	n.s.	
Hip RoM (°) S3		5.5 ± 5.3	2.2–8.7	7.7 ± 6.4	4.0–11.3	6.1 ± 9.1	1.0–11.1	n.s.	n.s.	
Hip RoM (°) S1	Female	6.9 ± 6.8	3.1–10.7	7.5 ± 5.5	4.4–10.7	5.7 ± 5.7	2.5–8.6	n.s.	n.s.	
Hip RoM (°) S2		4.3 ± 5.9	1.0–7.6	7.4 ± 8.6	2.4–12.3	6.6 ± 5.4	0.7–8.7	n.s.	n.s.	
Hip RoM (°) S3		6.3 ± 5.1	3.5–9.1	6.7 ± 6.3	3.1–10.3	4.7 ± 7.3	−1.8 to 7.6	n.s.	n.s.	
Knee RoM (°) S1	Male	−32.9 ± 4.6	−35.3 to −28.4	−37.4 ± 5.1	−39.7 to −33.6	−35.0 ± 8.9	−39.9 to −30.1	0.010 (VIS vs. CTRL) ES = 0.29	n.s.	n.s.
Knee RoM (°) S2		−32.6 ± 4.6	−35.6 to −25.4	−37.2 ± 6.4	−41.0 to −33.2	−37.1 ± 9.3	−42.2 to −31.9	0.034 (VIS vs. CTRL) ES = 0.61	n.s.	
Knee RoM (°) S3		−31.8 ± 6.4	−35.3 to −26.5	−36.6 ± 6.1	−39.8 to −32.4	−36.4 ± 8.4	−41.1 to −31.8	0.021 (VIS vs. CTRL) ES = 0.58	n.s.	
Knee RoM (°) S1	Female	−34.5 ± 6.5	−38.2 to −30.9	−34.6 ± 5.4	−37.6 to −31.6	−35.4 ± 5.0	−38.1 to −32.6	n.s.	0.022 (VER S1 vs. S3) ES = −0.48	
Knee RoM (°) S2		−32.7 ± 6.6	−36.3 to −29.0	−33.6 ± 6.1	−37.0 to −30.2	−33.6 ± 6.1	−36.9 to −30.2	n.s.		
Knee RoM (°) S3		−32.8 ± 7.8	−37.1 to −28.5	−34.0 ± 6.3	−37.5 to −30.5	−32.4 ± 7.1	−36.3 to −28.5	n.s.		
Ankle RoM (°) S1	Male	21.5 ± 12.0	14.6–28.5	18.8 ± 16.0	10.0–27.7	17.3 ± 14.3	9.4–25.2	n.s.	n.s.	n.s.
Ankle RoM (°) S2		25.3 ± 10.7	19.1–31.5	21.0 ± 17.1	11.5–30.5	18.9 ± 16.5	9.7–28.0	n.s.	n.s.	
Ankle RoM (°) S3		24.6 ± 10.9	18.3–30.9	19.3 ± 18.6	9.0–29.6	21.7 ± 16.6	12.5–30.9	n.s.	n.s.	
Ankle RoM (°) S1	Female	17.9 ± 13.4	10.2–25.6	15.6 ± 12.3	8.8–22.4	24.1 ± 12.7	17.1–31.1	n.s.	n.s.	
Ankle RoM (°) S2		18.1 ± 14.4	9.8–26.5	17.0 ± 13.9	9.3–24.6	25.6 ± 13.3	18.3–33.0	n.s.	n.s.	
Ankle RoM (°) S3		21.3 ± 18.0	10.9–31.6	19.6 ± 16.4	10.6–28.7	26.7 ± 12.9	19.5–33.8	n.s.	n.s.	

*VIS* visual group, *VER* verbal group, *CTRL* control group, *SD* standard deviation, *CI* confidence interval, *S1* session 1, *S2* session 2, *S3* session 3, *ES* effect size, *n.s.* not significant, *RoM* range of motion, *vs.* versus

**Table 2:** Results of repeated measures ANOVA for the dependent kinetic variables

Kinetic variables	Sex	CTRL		VIS		VER		*p* value group	*p* value time	*p* value sex
		Mean ± SD	95 % CI	Mean ± SD	95 % CI	Mean ± SD	95 % CI			
vGRF (N/kg) S1	Male	21.7 ± 3.3	19.8–23.6	25.2 ± 3.1	23.6–27.1	20.6 ± 3.8	18.5–22.7	0.002 (VIS vs. CTRL) ES = 1.14 0.003 (VIS vs. VER) ES = 1.39	n.s.	<0.001 (VIS) ES = −2.23
vGRF (N/kg) S2		22.0 ± 4.0	19.7–24.3	26.4 ± 2.9	24.2–27.4	20.8 ± 3.7	18.7–22.8	0.003 (VIS vs. CTRL) ES = 1.08 0.002 (VIS vs. VER) ES = 1.49	n.s.	
vGRF (N/kg) S3		22.5 ± 3.1	20.7–24.2	26.3 ± 3.2	23.4–26.9	20.6 ± 3.5	18.6–22.5	0.008 (VIS vs. CTRL) ES = 0.88 0.002 (VIS vs. VER) ES = 1.38	n.s.	
vGRF (N/kg) S1	Female	20.6 ± 2.9	19.0–22.2	18.3 ± 2.6	16.8–19.7	19.3 ± 3.3	17.5–21.1	0.023 (VIS vs. CTRL) ES = −0.81	n.s.	
vGRF (N/kg) S2		21.0 ± 2.2	19.8–22.2	18.5 ± 3.5	16.5–20.4	19.3 ± 4.0	17.2–21.5	0.023 (VIS vs. CTRL) ES = −1.15	n.s.	
vGRF (N/kg) S3		21.3 ± 3.3	19.5–23.2	18.8 ± 3.4	16.9–20.7	19.6 ± 3.7	17.5–21.6	0.023 (VIS vs. CTRL) ES = 0.78	n.s.	
Knee flexion moment (Nm/kg) S1	Male	−2.7 ± 0.7	−3.1 to −2.3	−3.7 ± 0.9	−4.3 to −3.3	−2.8 ± 0.6	−3.1 to −2.5	0.001 (VIS vs. CTRL) ES = −1.43 0.005 (VIS vs. VER) ES = −1.33	n.s.	<0.001 (VIS) ES = 1.72
Knee flexion moment (Nm/kg) S2		−2.8 ± 0.7	−3.1 to −2.4	−3.8 ± 1.2	−4.7 to −3.3	−3.0 ± 0.8	−3.5 to −2.6	0.009 (VIS vs. CTRL) ES = −1.28 0.021 (VIS vs. VER) ES = −0.94	n.s.	
Knee flexion moment (Nm/kg) S3		−2.9 ± 0.5	−3.2 to −2.7	−3.9 ± 1.3	−4.6 to −3.2	−2.9 ± 0.9	−3.4 to −2.5	0.011 (VIS vs. CTRL) ES = −1.04 0.049 (VIS vs. VER) ES = −0.89	n.s.	
Knee flexion moment (Nm/kg) S1	Female	−2.8 ± 0.6	−3.1 to −2.5	−2.4 ± 0.5	−2.7 to −2.1	−2.5 ± 0.7	−3.0 to −2.1	n.s.	n.s.	
Knee flexion moment (Nm/kg) S2		−2.8 ± 0.5	−3.0 to −2.5	−2.5 ± 0.5	−2.7 to −2.2	−2.5 ± 0.8	−2.9 to −2.0	n.s.	n.s.	
Knee flexion moment (Nm/kg) S3		−2.7 ± 0.5	−3.0 to −2.4	−2.4 ± 0.5	−2.6 to −2.1	−2.7 ± 0.8	−3.1 to −2.2	n.s.	n.s.	
Knee varus (+)/valgus (−) moment (Nm/kg) S1	Male	−0.23 ± 0.69	−0.63 to 0.16	−0.23 ± 0.78	−0.67 to 0.20	−0.21 ± 0.65	−0.57 to 0.15	n.s.	n.s.	0.040 (CTRL) ES = −0.86
Knee varus (+)/valgus (−) moment (Nm/kg) S2		−0.29 ± 0.69	−0.69 to 0.11	−0.12 ± 0.94	−0.64 to 0.40	−0.13 ± 0.62	−0.48 to 0.21	n.s.	n.s.	
Knee varus (+)/valgus (−) moment (Nm/kg) S3		−0.23 ± 0.69	−0.63 to 0.16	−0.06 ± 0.94	−0.58 to 0.46	−0.17 ± 0.61	−0.51 to 0.17	n.s.	n.s.	
Knee varus (+)/valgus (−) moment (Nm/kg) S1	Female	0.27 ± 0.48	−0.01 to 0.55	0.23 ± 0.50	−0.04 to 0.51	0.07 ± 0.43	−0.16 to 0.31	n.s.	n.s.	
Knee varus (+)/valgus (−) moment (Nm/kg) S2		0.31 ± 0.50	0.03–0.60	0.21 ± 0.46	−0.05 to 0.46	0.01 ± 0.38	−0.20 to 0.22	n.s.	n.s.	
Knee varus (+)/valgus (−) moment (Nm/kg) S3		0.18 ± 0.38	−0.03 to 0.40	0.25 ± 0.57	−0.07 to 0.56	−0.01 ± 0.33	−0.19 to 0.17	n.s.	n.s.	
Ankle dorsiflexion moment (Nm/kg) S1	Male	2.8 ± 0.7	2.5–3.2	3.1 ± 0.7	2.7–3.5	2.4 ± 0.7	1.2–2.8	0.022 (VIS vs. VER) ES = −0.92	0.050 (VIS S1 vs. S3) ES = 0.45 0.023 (VIS S2 vs. S3) ES = 0.51	0.035 (CTRL) ES = 0.76 <0.001 (VIS) ES = 1.35
Ankle dorsiflexion moment (Nm/kg) S2		2.8 ± 0.9	2.3–3.3	3.11 ± 0.8	2.7–3.6	2.4 ± 0.8	2.0–2.8	0.010 (VIS vs. VER) ES = −0.97		
Ankle dorsiflexion moment (Nm/kg) S3		3.0 ± 0.9	2.5–3.5	2.7 ± 0.8	2.3–3.2	2.5 ± 0.6	2.1–2.8	n.s.		
Ankle dorsiflexion moment (Nm/kg) S1	Female	2.2 ± 0.6	1.8–2.5	2.0 ± 0.5	1.7–2.3	2.3 ± 0.5	2.0–2.6	n.s.	n.s.	
Ankle dorsiflexion moment (Nm/kg) S2		2.2 ± 0.7	1.9–2.6	2.0 ± 0.7	1.6–2.4	2.4 ± 0.6	2.1–2.7	n.s.	n.s.	
Ankle dorsiflexion moment (Nm/kg) S3		2.5 ± 0.8	2.1–2.9	2.0 ± 0.7	1.6–2.4	2.4 ± 0.5	2.1–2.7	n.s.	n.s.	

*VIS* visual group, *VER* verbal group, *CTRL* control group, *SD* standard deviation, *CI* confidence interval, *vGRF* vertical ground reaction force, *S1* session 1, *S2* session 2, *S3* session 3, *ES* effect size, *n.s.* not significant, *vs.* versus

### Group comparison within sex

#### Kinematics

Males in the VIS group showed greater knee RoM compared to the males in the CTRL group in all sessions (*p* < 0.05). The males in the VIS group also showed greater ankle dorsiflexion angles compared to males in the CTRL group regardless of session (*p* < 0.05). Furthermore, the females in the VER group showed greater knee flexion angles compared to the females in the CTRL and VIS groups in each of the sessions (*p* < 0.05). Time-by-group interaction was found for trunk flexion angle in females: a decrease in the CTRL group and an increase in the VIS group (*p* = 0.001) (Table 1).

#### Kinetics

The males in the VIS group showed a significant larger mean vGRF in all sessions compared to the males in the VER and CTRL groups (*p* < 0.05). In addition, the females in the VIS group showed smaller mean vGRF compared to the females in the CTRL group, regardless of session (*p* < 0.05). The changes over time can also be seen in Fig. 1, where the male VIS group significantly increased vGRF from the start of S1 (24.1 Nm/kg) to the end of S1 (26.2 Nm/kg), *p* = 0.042. Furthermore, the males in the VIS group showed greater knee flexion moments compared to males in the VER and CTRL groups, regardless of sessions (*p* < 0.05). Additionally, the males in the VIS group showed reduction in their knee valgus moment over time, while the males in the VER and CTRL groups did not. On the other hand, the females in the VER group showed reduction in their knee varus moment over time, while the females in the CTRL group decreased in S3 after an increase in S2 (Fig. 1). These were, however, non-significant changes (Table 2).

### Sex comparison within group

#### Kinematics

In the VIS group, males showed greater knee flexion angles compared to the females (*p* = 0.047) (Table 1).

#### Kinetics

The males in the VIS group showed greater vGRF’s and greater knee flexion moments (*p* < 0.001) compared to the females in the VIS group, regardless of session (Fig. 2). In addition, males in the VIS group exhibited larger ankle dorsiflexion moments compared to the females in the VIS group in all sessions (*p* < 0.001). For the CTRL group, the males showed larger ankle dorsiflexion moments in all sessions compared to the females (*p* = 0.035) (Table 2).

## Discussion

The purpose of this study was to investigate the effects of EF (visual stimulus) and IF (verbal stimulus) on peak knee joint loading in female and male athletes during unexpected sidestep cutting and how these effects remained over time. Our hypothesis was partially confirmed: visual feedback led to reduced knee joint loading in male subjects with high retention.

### Effects of feedback

Overall, the major changes were observed within the male VIS group: they acquired better motor skills (S1) with retention (S2, S3). Their adopted landing strategy included larger vGRF [8.5 % increase in S1, *p* = 0.042 (Fig. 1)], knee flexion moment, knee RoM and ankle dorsiflexion angle. The soft landing strategy with larger knee RoM and ankle dorsiflexion angle allows for better load dissemination [12]. As the knee flexion angles were greater than 50°, along with a reduction in the knee valgus moment over time, this likely reduces combined loading of the ACL and the risk of ACL injury. Males in the VIS group seemed to be effective in performing the task by flexing their knee and absorbing mechanical energy while actively aligning the vGRF close to the centre of the knee joint (Fig. 2a). As the increased vGRF only had an impact on the sagittal knee moments, we feel this is not a major concern [33] and a reflection of increased motor control during this task. During heavy deceleration and a short contact time, there is no increase in frontal plane moment and the males use their quadriceps to execute the sidestep with a flexed knee to forcefully push off the force plate.

These findings are in agreement with others examining the effect of IF and EF instructions in healthy athletes, where the EF group increased knee flexion RoM during a counter movement jump [29]. Others have found a reduction in vGRF in double-legged jumps following video feedback [40, Dallinga et al. 2015, manuscript in review]; however, the task, sex, different modes or combinations of feedback may all contribute to the differences found. A direct comparison therefore cannot be made.

Frontal plane moments were less responsive to the VIS or VER feedback provided. In contrast, Dempsey et al. [14] noted reduced knee valgus moment following video feedback. These athletes saw both expert and own performances and received individual verbal feedback based upon required changes [14], and this combination of visual (expert and self) and verbal feedback could have positively influenced their results [43]. In double-legged jumps, knee valgus displacement was also positively influenced in two studies using visual feedback [17, 35], while no changes were found in another study [51]. Also providing subject views from different sides (front, side, back) may have resulted in better adoption of the required technique. With a complex manoeuvre such as sidestep cutting, more comprehensive feedback may be advantageous to achieve whole-body technique modification as knee load is also dependent on trunk control [20]. The instructions and feedback should be kept relatively simple though, as high complexity of feedback hampers motor learning [30].

### Sex differences

Of the two feedback techniques (VIS and VER), sex differences were only observed within the VIS group, with males showing larger knee flexion moments and vGRF compared to the females (Fig. 2). A potential explanation is that females use a ‘ligament dominant’ landing [36], which may place them at greater risk of ACL injury [26]. In addition, with reduced neuromuscular control, the direction of the vGRF is less controlled in all planes (Fig. 2b). The females in the VIS group did, however, increase trunk flexion angle over time, which is in favour for reducing potential ACL injury risk [19, 46].

It is not clear why the females in the current study were less responsive to feedback. Learning new strategies can be hampered in females if they use their muscles less effectively. Maybe females need more time to adopt a safe landing strategy. Additionally, the females in our study can be classified as ‘low-risk’ females (based on knee valgus moment), who have been shown to have smaller potential to change their movement pattern compared to ‘high-risk’ females [37]. Moreover, it is plausible that females in general prefer different learning strategies (e.g. combination of visual and verbal feedback) [24], considering the lack of effect in the females in the VIS group and the stronger effect in the female VER group for some of the variables (Fig. 1). Adding verbal instructions could enhance motor learning in females. However, literature shows that verbal instructions that induce an EF yield better results in motor skill acquisition and retention compared to IF instructions [54]. In another study of this research group, subjects performed a drop vertical jump with self-controlled expert video feedback, demonstrating beneficial results of verbal EF feedback in females and video feedback in females and males on landing technique, with one week retention (Welling et al. 2015, manuscript in review).

One group received instructions (VER, no change in wording), while the other group received feedback (VIS, change in video). This disparity could have affected motivation and therefore learning effect. Experiencing competence through good performance (knowing that you are watching your best trial, being involved in analysing own performance) adds to the evidence of motivational influences on motor learning (e.g. intrinsic motivation, interest and enjoyment, positive comparative feedback) [10]. Also, we did not examine how exactly subjects adhered to the instructions provided. Findings of this study may be limited to this specific population of recreational athletes and may not translate to other populations such as elite or younger athletes. Furthermore, the accuracy of skin-based markers in estimating joint kinematics and kinetics has been questioned [44].

Lastly, when considering the benchmarks from Cohen referred to in this manuscript, especially for the kinetic variables, most effect sizes are large. However, this is caused by the small standard deviations. Our group was homogeneous, and data collection was very stable and therefore reliable. The differences of the means do have a clinical relevance as indicated in the discussion, i.e. the changes depicted in the males receiving visual feedback reflect a potential safer landing strategy.

More evidence is needed regarding biomechanical risk factors for ACL injury in male athletes [2]. Future research should also examine the use of combinations of self-feedback (reviewing own video) and expert modelling (reviewing video from an expert), along with providing feedback ‘on demand’ (self-controlled learning) with verbal and visual cues. Future research should also focus on transfer to the field to examine whether the learned movement techniques remain during a practice or game. It is hypothesized that learned skills with an EF transfer better to the field [6].

Sex-specific learning preferences should be acknowledged when implementing ACL injury prevention programmes as males responded well to visual feedback, while females did not. For injury prevention, a safer landing technique as well as stable or increased performance is crucial [23]. The results of the current study and others [14] support the use of EF feedback. ACL injury risk may be reduced in males by an increase in knee flexion moment and decrease in knee valgus moment, respectively, whereas performance (running speed) was not compromised. These feedback techniques seem to be advantageous in contrary to IF verbal instructions leading to a decrease in performance (i.e. decreased jump height or movement speed) [13, 28, 49, 52].

The goal of ACL injury prevention is to achieve long-term effects and transfer learned skills to actual competition. None of the previous mentioned studies measured retention to examine whether a permanent change was achieved. In our study, retention was achieved after one and 4 weeks in the males in the VIS group as they continued to demonstrate superior technique. This indicates that the effect was not only immediate and temporary but also relatively permanent. Apparently, the learning process initiated with the visual feedback continues by repeating motor patterns in the brain even if no feedback is given (motor imagery) [55]. This is a very interesting given as this implicates that learning with a visual component stimulates automaticity and retention and therefore might require less time and investment from training staff. Recently, a single 15-min video feedback protocol led to greater reduction in dynamic knee valgus during a drop vertical jump task [35] than that has been observed after a four-week jump training programme [18].

The ‘whole-body approach’, that enhances being embedded in the task (embodied cognition), provided by visual feedback appears to be an effective method to promote motor learning [14]. Imitation plays an important role when receiving visual input [6]. The mirror neurons facilitate motor learning by automatically mapping observed movements onto a motor programme without high cognitive involvement [45].

Overall, the results of the present study demonstrate that learning through observation and practice is a powerful tool. The current study expands on the previous published conceptual and practical framework to enhance effectiveness of current ACL injury prevention programmes [4, 6]. Cumulatively, evidence is emerging that implementation of EF feedback especially with a visual feedback component is promising in terms of reducing ACL injury risk while maintaining or increasing performance.

In conclusion, sex-specific learning preferences may have to be acknowledged in day by day practice. Adding video instruction or feedback to regular training regimens when teaching athletes safe movement patterns and providing individual feedback might target suboptimal long-term results and optimize ACL injury prevention programmes.

## Conclusion

The current study shows that male subjects clearly benefit from visual feedback. Females may need different feedback modes to optimize their sidestep cutting technique. Future research is needed to examine the transfer to the field to investigate whether the learned movement techniques remain during a practice or game.

## References

[1] Aerts I, Cumps E, Verhagen E, Wuyts B, Van De Gucht S, Meeusen R (2015). The effect of a 3-month prevention program on the jump-landing technique in basketball: a randomized controlled trial. J Sport Rehabil.

[2] Alentorn-Geli E, Mendiguchia J, Samuelsson K, Musahl V, Karlsson J, Cugat R, Myer GD (2014). Prevention of anterior cruciate ligament injuries in sports. Part I: systematic review of risk factors in male athletes. Knee Surg Sports Traumatol Arthrosc.

[3] Beaulieu M, Lamontagne M, Xu L (2008). Gender differences in time-frequency EMG analysis of unanticipated cutting maneuvers. Med Sci Sports Exerc.

[4] Benjaminse A, Gokeler A, Dowling AV, Faigenbaum A, Ford KR, Hewett TE, Onate JA, Otten B, Myer GD (2015). Optimization of the anterior cruciate ligament injury prevention paradigm: novel feedback techniques to enhance motor learning and reduce injury risk. J Orthop Sports Phys Ther.

[5] Benjaminse A, Gokeler A, Fleisig GS, Sell TC, Otten B (2011). What is the true evidence for gender-related differences during plant and cut maneuvers? A systematic review. Knee Surg Sports Traumatol Arthrosc.

[6] Benjaminse A, Otten E (2011). ACL injury prevention, more effective with a different way of motor learning?. Knee Surg Sports Traumatol Arthrosc.

[7] Benjaminse A, Welling W, Otten B, Gokeler A (2015). Novel methods of instruction in ACL injury prevention programs, a systematic review. Phys Ther Sport.

[8] Celebrini RG, Eng JJ, Miller WC, Ekegren CL, Johnston JD, Depew TA, Macintyre DL (2014). Effect of a novel movement strategy in decreasing ACL risk factors in female adolescent soccer players: a randomized controlled trial. Clin J Sport Med.

[9] Celebrini RG, Eng JJ, Miller WC, Ekegren CL, Johnston JD, MacIntyre DL (2012). The effect of a novel movement strategy in decreasing ACL risk factors in female adolescent soccer players. J Strength Cond Res.

[10] Chiviacowsky S, Wulf G, Lewthwaite R (2012). Self-controlled learning: the importance of protecting perceptions of competence. Front Psychol.

[11] Cohen J (2013). Statistical power analysis for the behavioral science.

[12] Dai B, Butler RJ, Garrett WE, Queen RM (2014). Using ground reaction force to predict knee kinetic asymmetry following anterior cruciate ligament reconstruction. Scand J Med Sci Sports.

[13] Dai B, Garrett WE, Gross MT, Padua DA, Queen RM, Yu B (2015). The effects of 2 landing techniques on knee kinematics, kinetics, and performance during stop-jump and side-cutting tasks. Am J Sports Med.

[14] Dempsey AR, Lloyd DG, Elliott BC, Steele JR, Munro BJ (2009). Changing sidestep cutting technique reduces knee valgus loading. Am J Sports Med.

[15] Donnelly CJ, Elliott BC, Ackland TR, Doyle TL, Beiser TF, Finch CF, Cochrane JL, Dempsey AR, Lloyd DG (2012). An anterior cruciate ligament injury prevention framework: incorporating the recent evidence. Res Sports Med.

[16] Hagglund M, Atroshi I, Wagner P, Walden M (2013). Superior compliance with a neuromuscular training programme is associated with fewer ACL injuries and fewer acute knee injuries in female adolescent football players: secondary analysis of an RCT. Br J Sports Med.

[17] Herman DC, Onate JA, Weinhold PS, Guskiewicz KM, Garrett WE, Yu B, Padua DA (2009). The effects of feedback with and without strength training on lower extremity biomechanics. Am J Sports Med.

[18] Herrington L (2010). The effects of 4 weeks of jump training on landing knee valgus and crossover hop performance in female basketball players. J Strength Cond Res.

[19] Hewett TE, Torg JS, Boden BP (2009). Video analysis of trunk and knee motion during non-contact anterior cruciate ligament injury in female athletes: lateral trunk and knee abduction motion are combined components of the injury mechanism. Br J Sports Med.

[20] Jamison ST, McNally MP, Schmitt LC, Chaudhari AM (2013). The effects of core muscle activation on dynamic trunk position and knee abduction moments: implications for ACL injury. J Biomech.

[21] Joy EA, Taylor JR, Novak MA, Chen M, Fink BP, Porucznik CA (2013). Factors influencing the implementation of anterior cruciate ligament injury prevention strategies by girls soccer coaches. J Strength Cond Res.

[22] Kadaba MP, Ramakrishnan HK, Wootten ME, Gainey J, Gorton G, Cochran GV (1989). Repeatability of kinematic, kinetic, and electromyographic data in normal adult gait. J Orthop Res.

[23] Keats MR, Emery CA, Finch CF (2012). Are we having fun yet? Fostering adherence to injury preventive exercise recommendations in young athletes. Sports Med.

[24] Kimura D (2004). Human sex differences in cognition, fact, not predicament. Sex Evol Gender.

[25] Kristianslund E, Krosshaug T (2013). Comparison of drop jumps and sport-specific sidestep cutting: implications for anterior cruciate ligament injury risk screening. Am J Sports Med.

[26] Laughlin WA, Weinhandl JT, Kernozek TW, Cobb SC, Keenan KG, O’Connor KM (2011). The effects of single-leg landing technique on ACL loading. J Biomech.

[27] Lindblom H, Waldén M, Carlfjord S, Hägglund M (2014). Implementation of a neuromuscular training programme in female adolescent football: 3-year follow-up study after a randomised controlled trial. Br J Sports Med.

[28] Lindblom H, Waldén M, Hägglund M (2012). No effect on performance tests from a neuromuscular warm-up programme in youth female football: a randomised controlled trial. Knee Surg Sports Traumatol Arthrosc.

[29] Makaruk H, Porter JM, Czaplicki A, Sadowski J, Sacewicz T (2012). The role of attentional focus in plyometric training. J Sports Med Phys Fitness.

[30] Marchant DC, Clough PJ, Crawshaw M (2007). The effects of attentional focusing strategies on novice dart throwing performance and their task experiences. Int J of Sport Exerc Psych.

[31] McGinley JL, Baker R, Wolfe R, Morris ME (2009). The reliability of three-dimensional kinematic gait measurements: a systematic review. Gait Posture.

[32] McGlashan AJ, Finch CF (2010). The extent to which behavioural and social sciences theories and models are used in sport injury prevention research. Sports Med.

[33] McLean SG, Huang X, Su A, Van Den Bogert AJ (2004). Sagittal plane biomechanics cannot injure the ACL during sidestep cutting. Clin Biomech (Bristol, Avon).

[34] McLean SG, Huang X, van den Bogert AJ (2005). Association between lower extremity posture at contact and peak knee valgus moment during sidestepping: implications for ACL injury. Clin Biomech (Bristol, Avon).

[35] Munro A, Herrington L (2014). The effect of videotape augmented feedback on drop jump landing strategy: implications for anterior cruciate ligament and patellofemoral joint injury prevention. Knee.

[36] Myer GD, Brent JL, Ford KR, Hewett TE (2011). Real-time assessment and neuromuscular training feedback techniques to prevent ACL injury in female athletes. Strength Cond J.

[37] Myer GD, Ford KR, Brent JL, Hewett TE (2007). Differential neuromuscular training effects on ACL injury risk factors in “high-risk” versus “low-risk” athletes. BMC Musculoskelet Disord.

[38] Myklebust G, Engebretsen L, Braekken IH, Skjolberg A, Olsen OE, Bahr R (2003). Prevention of anterior cruciate ligament injuries in female team handball players: a prospective intervention study over three seasons. Clin J Sport Med.

[39] Olsen OE, Myklebust G, Engebretsen L, Holme I, Bahr R (2005). Exercises to prevent lower limb injuries in youth sports: cluster randomised controlled trial. BMJ.

[40] Onate JA, Guskiewicz KM, Sullivan RJ (2001). Augmented feedback reduces jump landing forces. J Orthop Sports Phys Ther.

[41] Pollard CD, Davis IM, Hamill J (2004). Influence of gender on hip and knee mechanics during a randomly cued cutting maneuver. Clin Biomech (Bristol, Avon).

[42] Pollard CD, Stearns KM, Hayes AT, Heiderscheit BC (2015). Altered lower extremity movement variability in female soccer players during side-step cutting after anterior cruciate ligament reconstruction. Am J Sports Med.

[43] Ram N, Riggs SM, Skaling SM, Landers DM, McCullagh P (2007). A comparison of modelling and imagery in the acquisition and retention of motor skills. J Sport Sci.

[44] Reinschmidt C, van den Bogert AJ, Nigg BM, Lundberg A, Murphy N (1997). Effect of skin movement on the analysis of skeletal knee joint motion during running. J Biomech.

[45] Rizzolatti G, Fabbri-Destro M, Cattaneo L (2009). Mirror neurons and their clinical relevance. Nat Clin Pract Neurol.

[46] Sasaki S, Nagano Y, Kaneko S, Imamura S, Koabayshi T, Fukubayashi T (2015). The relationships between the center of mass position and the trunk, hip, and knee kinematics in the sagittal plane: a pilot study on field-based video analysis for female soccer players. J Hum Kin.

[47] Schmidt RA, Lee T (2014). Motor learning and performance.

[48] Sigward SM, Powers CM (2006). The influence of gender on knee kinematics, kinetics and muscle activation patterns during side-step cutting. Clin Biomech (Bristol, Avon).

[49] Steffen K, Bakka HM, Myklebust G, Bahr R (2008). Performance aspects of an injury prevention program: a ten-week intervention in adolescent female football players. Scand J Med Sci Sports.

[50] Sugimoto D, Myer GD, McKeon JM, Hewett TE (2012). Evaluation of the effectiveness of neuromuscular training to reduce anterior cruciate ligament injury in female athletes: a critical review of relative risk reduction and numbers-needed-to-treat analyses. Br J Sports Med.

[51] Tate JJ, Milner CE, Fairbrother JT, Zhang S (2013). The effects of a home-based instructional program aimed at improving frontal plane knee biomechanics during a jump-landing task. J Orthop Sports Phys Ther.

[52] Vescovi JD, Rupf R, Brown TD, Marques MC (2011). Physical performance characteristics of high-level female soccer players 12–21 years of age. Scand J Med Sci Sports.

[53] Windolf M, Götzen N, Morlock M (2008). Systematic accuracy and precision analysis of video motion capturing systems–exemplified on the Vicon-460 system. J Biomech.

[54] Wulf G, McConnel N, Gartner M, Schwarz A (2002). Enhancing the learning of sport skills through external-focus feedback. J Mot Behav.

[55] Wulf G, Shea C, Lewthwaite R (2010). Motor skill learning and performance: a review of influential factors. Med Educ.

